# Brain‐responsive neurostimulation treatment in patients with GAD65 antibody–associated autoimmune mesial temporal lobe epilepsy

**DOI:** 10.1002/epi4.12395

**Published:** 2020-04-14

**Authors:** Anteneh M Feyissa, Emily A. Mirro, Angela Wabulya, William O. Tatum, Kaitlyn E. Wilmer‐Fierro, Hae Won Shin

**Affiliations:** ^1^ Department of Neurology Mayo Clinic Florida Jacksonville Florida; ^2^ NeuroPace, Inc. Mountain View California; ^3^ Department of Neurology University of North Carolina at Chapel Hill Chapel Hill North Carolina

**Keywords:** autoimmune epilepsy, brain‐responsive neurostimulation, drug‐resistant epilepsy, GAD65 antibody, temporal lobe epilepsy

## Abstract

Glutamic acid decarboxylase 65‐kilodalton isoform (GAD65) antibodies have been associated with multiple nonneurological and neurological syndromes including autoimmune epilepsy (AE). Although immunotherapy remains the cornerstone for the treatment of AE, those with GAD65 Ab‐associated AE (GAD65‐AE) remain refractory to immunotherapy and antiseizure medication (ASM). Outcomes of epilepsy surgery in this patient population have also been unsatisfactory. The role of neuromodulation therapy, particularly direct brain‐responsive neurostimulation therapy, has not been previously examined in GAD65‐AE. Here, we describe four consecutive patients with refractory GAD‐65‐associated temporal lobe epilepsy (GAD65‐TLE) receiving bilateral hippocampal RNS System treatment. The RNS System treatment was well tolerated and effective in this study cohort. Three patients had a >50% clinical seizure reduction, and one patient became clinically seizure‐free following resective surgery informed by the RNS System data with continued RNS System treatment. In all four of our patients, the long‐term ambulatory data provided by the RNS System allowed us to gain objective insights on electrographic seizure lateralization, patterns, and burden as well as guided immunotherapy and ASM optimization. Our results suggest the potential utility of the RNS System in the management of ASM intractable GAD65‐AE.

**Key point**

- RNS System treatment was well‐tolerated & effective in four patients with drug‐resistant GAD65 antibodyassociated temporal lobe epilepsy.
- RNS System treatment resulted in >50% seizure reduction in 3 patients; 1 is seizure‐free after RNS System data‐guided temporal lobectomy.
- RNS System ECoGs provided insight on seizure lateralization, patterns, and seizure burden, and guided immunotherapy and ASM optimization.

## 1 INTRODUCTION

Autoimmune epilepsy (AE) is an immunologically mediated disorder in which recurrent seizures are a persistent clinical feature.^1^ Autoimmune origin is confirmed by the chronic presence of antibody to neural proteins or the demonstration of chronic brain inflammation.^1^, ^2^, ^3^ Up to 20% of epilepsies of unknown etiology may be due to AE.^2^, ^3^ The most commonly identified antibodies are those targeting N‐methyl‐d‐aspartate receptor (NMDAR), leucine‐rich glioma‐inactivated protein 1 (LGI1), and glutamic acid decarboxylase 65 (GAD65).^3^ GAD65 antibody–associated AE (GAD65‐AE) is a rare but distinct neurological syndrome with a wide clinical spectrum ranging from mild nonpharmacoresistant epilepsy^4^ to drug‐resistant temporal lobe epilepsy (GAD65‐TLE),^5^ limbic encephalitis,^6^ and extra‐limbic encephalitis.^5^, ^7^ Ten percent of chronic epilepsy patients are estimated to harbor GAD65 antibodies.^5^

Although immunotherapies have demonstrated efficacy in the management of patients with cell‐surface antibody‐associated AEs (eg, LGI1), the response of GAD65‐AE to immunotherapy is poor, with few patients achieving seizure‐freedom.^8^ Antiseizure medication (ASM) management has demonstrated efficacy in <10% patients.^9^ Seizure reductions after epilepsy surgery are significantly lower than for other etiologies, including unilateral mesial temporal sclerosis (MTS).^10^ There are reports of vagus nerve stimulation (VNS) therapy for the treatment of drug‐resistant focal AE^11^, but no previous report of direct brain‐responsive neurostimulation (RNS^®^ System, NeuroPace, Inc) treatment for AE. This paper describes four GAD65‐TLE cases successfully treated with the RNS System.

## 2 METHODS

After protocol approval by the participating institutions' institutional review board, subjects gave written informed consent. Subsequently, a retrospective chart review was completed for four drug‐resistant GAD65‐AE patients treated with the RNS System at Mayo Clinic Florida or University of North Carolina (Chapel Hill). Data collection (from electronic medical records (EMRs) and the NeuroPace Patient Data Management System data repository) included demographics, seizure history, history of ASM and immunotherapy, presurgical evaluation including brain imaging, scalp video‐electroencephalogram (VEEG), intracranial video‐electroencephalogram (iEEG) when applicable, RNS System implant information, long‐term ambulatory electrocorticograms (RNS System ECoGs), clinical response to RNS System treatment at 12 months and most recent follow‐up, and serious adverse events. Of note, during the course of their illness all patients underwent serum or CSF antibody epilepsy panel testing including AchR ganglionic neuronal, AGNA‐1, AMPA‐R, amphiphysin, ANNA‐1, ANNA‐2, ANNA‐3, CASPR2, CRMP‐5, GABA‐B, GAD65, LGI1, NMDA, mGluR1, N‐type calcium channel, P/Q‐type calcium channel, PCA‐2, and PCA‐Tr antibodies. Anti‐GAD65 antibody was measured using double‐antibody radioimmunoassay.

## 3 RESULTS

Four patients with drug‐resistant GAD65‐AE underwent RNS System treatment with bilateral hippocampal depth leads. Patients resemble previous populations with GAD65‐AE,^4^, ^5^, ^6^, ^7^ all female and young (mean = 28 years; range = 21‐37 years). No RNS System postoperative complications and no stimulation‐related adverse effects were reported. Clinical characteristics, seizure outcome, and RNS System stimulation parameters at last follow‐up are summarized (Table 1).

**TABLE 1:** Demographics, seizure characteristics, and clinical outcomes

	Case#1	Case#2	Case#3	Case#4
Age (years)	37	27	28	21
Sex	Female	Female	Female	Female
Seizure type(s)	FAS and FIAS	FIAS and GTCs	FAS and FIAS	FAS and FIAS
GAD65 Antibody titer (nmol/L)	Serum:252 CSF:3.5	Serum: 4538 CSF: 8.61	CSF: 0.39	Serum: 105
# ASM trials	9	6	3	7
Immunotherapy trials	IVIG and IVMP	IVIG, Mycophenolate, and Prednisone	Prednisone	IVIG, PLEX, Rituximab, and Prednisolone
*Presurgical evaluation*				
EMU, seizures	Independent bitemporal	Bilateral frontotemporal	Independent bitemporal	Diffuse
MRI	Left MTS	Right MTS	Left MTS	Right MTS
PET	Bitemporal hypometabolism	None‐localizing	None‐localizing	NA
Ictal SPECT	Right temporal hyperperfusion	Right temporal hyperperfusion	Right temporal hyperperfusion	NA
iEEG, seizures	NA	independent bilateral hippocampal	Left Temporal	Independent bilateral hippocampal
Pre‐RNS System clinical seizure rate	42‐56 per week	>4 per week	21‐28 per week	56‐70 per week
Post‐RNS System clinical seizure reduction at last follow‐up	>75% reduction of right onset >50% reduction of left onset	75% reduction of right and left onset	Free of clinical seizures	50%–75% reduction of right and left onset
Length of follow‐up (months)	27	33	24	15
RNS System parameter setting at last follow‐up	*Burst 1*: Monopolar cathodal on Lead 2 (Right), 7.0 mA, 5 Hz, 160 µs, 2000 ms (3.5 µC/cm^2^) *Burst 2*: Monopolar cathodal on Lead 1 (Left), 7.0 mA, 5 Hz, 160 µs, 2000 ms (3.5 µC/cm^2^)	*Burst 1*: Grouped bipolar on Lead 1 (Left), 3.2 mA, 200 Hz, 200 µs, 100 ms (4.1 µC/cm^2^) *Burst 2*: Grouped bipolar on Lead 2 (Right), 3.2 mA, 200 Hz, 200 µs, 100 ms (4.1 µC/cm^2^)	*Burst 1*: Monopolar cathodal on Lead 2 (Right), 6.5 mA, 200 Hz, 200 µs, 100 ms (4.1 µC/cm^2^) *Burst 2*: Monopolar cathodal on Lead 1 (Left), 3.5 mA, 100 Hz, 160 µs, 100 ms (1.8 µC/cm^2^)	*Burst 1*: Bipolar on Lead 1 (Left), 2.5 mA, 200 Hz, 160 µs, 100 ms, (2.5 µC/cm^2^) *Burst 2*: Bipolar on Lead 2 (Right), 2.5 mA, 200 Hz, 160 µs, 100 ms, (2.5 µC/cm^2^)
Other comments	RNS System data identified increased seizure burden at night (led to ASM adjustments) RNS System data also revealed partial immunotherapy response	RNS System data established that >75% of seizures arose from left hippocampus (potential resection target)	RNS System data established that 90% of seizures arose from left hippocampus that led to left ATL and patient becoming free of clinical seizures	RNS System data established that 90% of seizures arose from left hippocampus (potential resection target)

Abbreviations: ASM, antiseizure medication; ATL, anterior temporal lobectomy; EMU, epilepsy monitoring unit; FAS, focal aware seizures; FIAS, focal impaired aware seizures; GAD65; glutamic acid decarboxylase 65‐kilodalton isoform; GTCs, generalized tonic‐clonic seizures; iEEG, intracranial electroencephalogram; IVIG, intravenous immunoglobulin; IVMP, intravenous methylprednisolone; MTS, mesial temporal sclerosis; NA, not applicable; PLEX, plasma exchange.

### 3.1 Case#1

A 37‐year‐old female, with a history of two brief typical febrile seizures and head injuries, had a 10‐year history of refractory epilepsy, suspected to be related to GAD65‐Ab (CSF = 3.5 nmol/L; serum = 252 nmol/L). Seizure semiology consisted of sharp distressful left‐sided chest pain with autonomic features with/without impairment of awareness lasting 1‐2 minutes. Despite nine ASM trials and immunotherapy (intravenous immunoglobulin (IVIG), intravenous methylprednisolone (IVMP)), seizure rate was 6‐8 seizures/d. Brain MRI showed left MTS and fluorodeoxyglucose positron emission tomography (PET) showed bitemporal hypometabolism. Interictal VEEG revealed independent bitemporal discharges and ictal VEEG captured independent bitemporal onsets. Ictal single‐photon emission computed tomography (SPECT) showed right temporal hyperperfusion. Subsequently, she underwent RNS System implantation with bilateral hippocampal leads.

**FIGURE 1:**
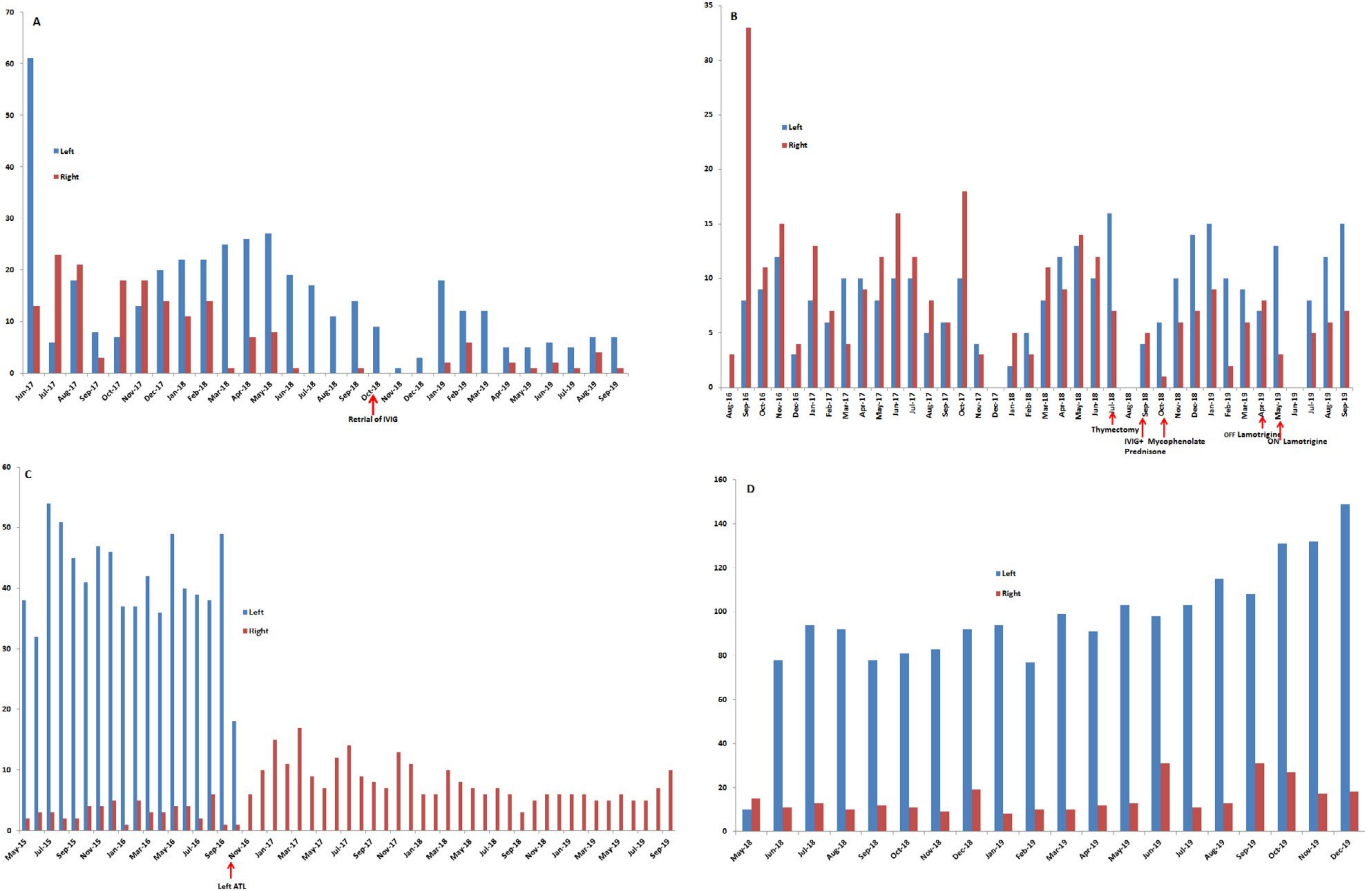
Count of electrographic seizure activity plotted over time for four patients from this study cohort, GAD65 temporal lobe epilepsy following RNS System treatment with bilateral hippocampal leads. The bars (*y*‐axis) represent long episode detections from lead electrode contacts overlying the right (red) and left (blue) mesial temporal lobe. In most cases, these represent electrographic seizures. A, shows favorable response to intravenous immunoglobulin (IVIG) [Case#1]. B, shows fluctuation of electrographic seizure lateralization by antiseizure medication therapy status (lamotrigine) [Case#2]. C, shows response following left anterior temporal lobectomy (ATL) guided by RNS System data [Case#3]. D, shows a majority of long episodes (>90%) arising from the left hippocampus; this trend is being monitored for a potential curative left ATL [Case#4]

After 27 months of RNS System treatment, the patient experienced substantial electrographic seizure reduction, >80% on the right and >50% on the left (Figure 1A). A repeat trial of IVIG immunotherapy 15 months following RNS System implantation showed improvement in seizure burden. Clinical seizures became significantly shorter and less severe/painful. Neuropsychiatric evaluation revealed an improvement in verbal memory. RNS System ECoGs helped establish nocturnal seizure clustering, allowing for ASM modification. Further, the RNS System helped establish the role of IVIG infusion (Figure 1A).

### 3.2 Case#2

A 27‐year‐old female had a 10‐year history of focal impaired awareness seizures and generalized tonic‐clonic seizures. Despite six ASM trials, seizure rate was >4 seizures/wk. Brain MRI revealed right MTS. Inpatient VEEG recorded bilateral frontotemporal seizures. Ictal SPECT showed increased right temporal and parietal hyperperfusion, while PET‐MRI was nonlocalizing. Inpatient iEEG monitoring, with strip electrodes over the bilateral temporal (anterior, posterior, basal) and frontoparietal regions and bilateral depth electrodes in the amygdala and hippocampi, showed frequent interictal epileptiform discharges in the both hippocampi and anterior temporal regions. Stereotypical seizures independently arising from bilateral hippocampi were captured. Most clinical seizures arose from the left hippocampus, while most subclinical seizures arose from the right hippocampus. Subsequently, the patient underwent RNS System implantation with bilateral hippocampal leads.

Initial response to RNS System treatment was favorable; however, after 25 months the patient developed myasthenia gravis. Following treatment with pyridostigmine and thymectomy, seizure frequency was further reduced. Serum and CSF autoimmune epilepsy panel were positive for anti‐GAD 65‐Ab (CSF = 8.61 nmol/L; serum = 4538 nmol/L). Immunotherapy (IVIG, mycophenolate, prednisone) was unsuccessful. RNS System ECoGs revealed that over time the electrographic seizure onset lateralized from predominantly right to predominantly left (>75%). The latter trend is being monitored for a potential palliative left anterior temporal lobectomy (ATL) in the future. Interestingly, upon inadvertently missing a ASM dose (lamotrigine), electrographic seizure lateralization returned to predominantly right; however, upon resuming full ASM regimen, electrographic seizure lateralization became predominantly left (Figure 1B).

### 3.3 Case#3

A 28‐year‐old female with a past medical history of depression and Hashimoto thyroiditis had a 10‐year history of focal aware seizures and focal impaired awareness seizures. Despite three ASM trials, her seizure rate was 3‐4 seizures/d. MRI revealed evidence of left MTS. Inpatient VEEG recorded independent bitemporal onsets. Ictal SPECT showed increased right temporal hyperperfusion, whereas PET‐MRI was nonlocalizing. Antibody screening revealed abnormal thyroid peroxidase. CSF autoimmune antibody panel was positive for GAD65 antibody (CSF = 0.39 nmol/L). Immunotherapy (IVMP) was unsuccessful. Inpatient iEEG monitoring, with strip electrodes over the bilateral temporal (anterior, posterior, basal) and frontoparietal regions and bilateral depth electrodes in the amygdala and hippocampi, showed frequent interictal epileptiform discharges in the bilateral hippocampi and anterior temporal regions. Nine electrographic seizures arising from the left hippocampus were captured. Subsequently, the patient underwent RNS System implantation with bilateral hippocampal leads.

After nine months of RNS System treatment, the patient reported significant clinical seizure reduction; however, at 17 months reported no clinical seizure reduction (returned to preimplant baseline). RNS System ECoGs revealed that >90% of electrographic seizures arose from the left, leading to a successful left ATL. After 24 months of RNS System treatment, the patient continued to have no clinical seizures, and infrequent right electrographic seizures, with continued right‐sided RNS System treatment (Figure 1C).

### 3.4 Case#4

A 21‐year‐old female had a 5‐year history of focal aware and focal impaired awareness seizures. Despite seven ASM trials, her seizure rate was 8‐10 seizures/d. MRI revealed right MTS, while inpatient VEEG monitoring recorded bilateral diffuse onsets. Autoimmune epilepsy panel detected anti‐GAD 65‐Ab (serum = 105 nmol/L). Immunotherapy (IVIG, plasma exchange, mycophenolate, rituximab) was unsuccessful. Inpatient iEEG monitoring (using stereoelectroencephalography), with strip electrodes over the bilateral temporal (anterior, posterior, subtemporal) and frontoparietal regions and bilateral depth electrodes in the amygdala and hippocampi, showed frequent interictal epileptiform discharges arising from the bilateral hippocampal and anterior subtemporal regions. Numerous electrographic seizures independently arising from bilateral hippocampi (right > left) were captured. Eventually, the patient underwent RNS System implantation with bilateral hippocampal leads.

After 15 months of RNS System treatment, the patient reported 50%–75% clinical seizure reduction. RNS System ECoGs did not show a significant reduction in electrographic seizures, however did establish that 90% of electrographic seizures originated on the left (Figure 1D). This trend is being monitored for a potential curative left ATL.

## 4 DISCUSSION

This case series describes the clinical utility of the RNS System in the management of drug‐resistant GAD65‐TLE. A majority of this study cohort had >50% seizure reduction; one patient became seizure‐free following RNS System data‐guided resective surgery with continued RNS System treatment. RNS System ECoGs provided insight on electrographic seizure lateralization, patterns, and seizure burden, and guided immunotherapy and ASM optimization. No patient had surgery or device‐related complications.

Most patients with GAD65‐AE follow a chronic course, with low likelihood of long‐term seizure‐freedom. Management of GAD65‐TLE has been based on small case reports and treatment results with ASM, immunotherapy, and epilepsy surgery. One patient in this study cohort showed partial response to immunotherapy (Case#1), and none responded favorably to ASM alone. The lack of response to immunotherapy could be due to disease duration and delayed initiation of immunotherapy. Although early immunotherapy is advocated in GAD65‐AE, immunotherapy response is less than observed in cell‐surface antibody‐associated AEs.^12^ Epilepsy surgery has been previously performed for refractory cases; however, surgical outcomes were unsatisfactory.^10^

There are a few reports of VNS therapy in intractable AE.^11^ In addition to neuromodulation, VNS therapy has been speculated to have a modulatory effect on immune cell reactivity.^13^ The mechanisms mediating the effects of RNS System treatment on seizures may be due to depolarization blockade, synaptic inhibition, and long‐term pathologic network modulation.^14^ Currently, it is unknown whether direct brain‐responsive neurostimulation exerts a local modulatory effect on inflammatory mediators. It is possible that RNS System treatment may exert immunomodulatory effect similar to the anterior nucleus deep brain stimulation (ANT‐DBS), which has been shown to reverse hippocampal pro‐inflammatory state.^15^, ^16^ In Kainate‐induced seizures in rats, ANT‐DBS induced a normalized gene expression of pro‐inflammatory cytokines such as IL‐1β and IL‐6 and prevented subsequent neuronal injury in hippocampal CA1.^16^ If RNS System treatment was to have similar properties as ANT‐DBS, then modulation of neural activity could possibly counteract aberrant pro‐inflammatory responses present in AE. Nevertheless, the positive response to RNS System treatment in this study cohort (>50% seizure reduction) is similar to that reported in non–immune‐mediated drug‐resistant mesial TLE. During clinical trials, mesial TLE patients experienced a 70% median percent seizure reduction.^17^ Mesial TLE associated with GAD65‐Ab likely resulted from an ongoing inflammation process that persisted after the acute phase of encephalitis, or from irreversible changes that altered neuronal networks and persisted after the inflammatory process resolved.^18^

The RNS System provides data in a naturalistic setting and may complement inpatient iEEG data and contribute to treatment decisions.^19^ In this study cohort, the RNS System provided useful information about electrographic seizure lateralization, seizure timing and patterns, and response to certain ASM.^20^ Indeed, for one patient, ASM optimization was guided by RNS System ECoGs (Case#1); in another patient, seizure lateralization varied by ASM regimen (Case#2); and in a third patient, RNS System ECoGs facilitated a successful left ATL (Case#3).

Safety of the RNS System in focal epilepsy has been established in multiple clinical trials.^21^, ^22^, ^23^ The most frequent serious device‐related adverse event for the TLE cohort treated with the RNS System was soft tissue implant‐site infection (11.7%) and intracranial hemorrhage (2.7%), similar to other neurostimulation devices.^17^ Infections were primarily superficial soft tissue only and typically occurred in perioperative and acute postoperative periods. Device‐related infections generally required treatment with antibiotics and sometimes explantation. No patient in this study cohort had device‐related infection even though all patients received immunosuppressive drugs prior to and following RNS System implantation.

### 4.1 Study limitations

This study has inherent limitations. Data collection was based on extraction of patient information from EMRs, which was not documented in a structured, prospective fashion. For instance, seizure frequency and precise timing of drug effects were not routinely recorded, which may introduce confounding with multiple ASM and immunotherapies as well as concurrent RNS System treatment. The small sample size also limits any definitive conclusions regarding the role of RNS System treatment for drug‐resistant GAD65‐TLE. Selection bias could be present due to the tertiary nature of these patients. Lastly, seizure quantification prior to RNS System treatment was based on verbal report and therefore could be impacted by recall bias of amnestic seizures. Well‐designed future prospective studies are needed to establish the safety and efficacy of RNS System treatment in AE.

## 5 CONCLUSION

This small case series indicates that direct brain‐responsive neurostimulation may play a role in the management of drug‐resistant GAD65‐TLE. RNS System treatment could be considered when immunotherapy and ASMs do not accomplish seizure‐freedom. Future prospective studies are needed to validate the therapeutic utility and tolerability of the RNS System for drug‐resistant AE. Clinical studies are also required to elucidate whether RNS System treatment exerts immunomodulatory effects similar to that hypothesized for VNS and ANT‐DBS therapies.

## CONFLICT OF INTEREST

Author Emily A Mirro has equity ownership/stock options with NeuroPace and is an employee of NeuroPace. Author William O Tatum has received support from the Mayo Clinic, and Engage Pharmaceuticals, Liva Nova, Esai Pharmaceuticals, Martin Family Foundation, Elsevier, Demos Publishing, and Springer Publishers. Author Kaitlyn E Wilmer‐Fierro has equity ownership/stock options with NeuroPace and is an employee of NeuroPace. The remaining authors have no conflicts of interest. We confirm that we have read the Journal's position on issues involved in ethical publication and affirm that this report is consistent with those guidelines.
